# Bromide of Potassium

**Published:** 1869-04

**Authors:** George W. Hall

**Affiliations:** Prof. of Physiology, Pathology and Therapeutics, College of Physicians and Surgeons, Keokuk


### BROMIDE OF POTASSIUM.

### EXTRACT FROM A LECTURE.

BY GEORGE. W. HALL, M.D.,
Prof. of Physiology, Pathology and Therapeutics, College of Physicians and Surgeons, Keokuk.

1. Bromide of Potassium exerts an anæsthetic influence over all the mucus membranes, and more especially over the mucus membrane of the larynx and pharynx. It can be used therefore in preparing persons for laryngoscopic examination. Waring refers to the case of a man whose eye had been injured by a pistol shot. Under the influence of the bromide the conjunctinæ in this case became so insensible to pain that a portion of the membrane was removed, and particles of powder extracted without the least suffering. From fifteen to thirty grains, repeated every hour till two or three doses are given, are required to produce this effect This effect of the bromide —anæsthesia of the mucus membranes—renders it a useful remedy in pneumonia, bronchitis, pertursis, and other conditions attended with cough.

It will not "cure" or cut short whooping cough, as some have alleged. It only relieves the severity of the cough.

2. The bromide of potassium will allay "irritation" of the nervous system. In chorea, not depending upon congestion of the spinal cord or some portion of the brain, the bromide will aid in controling the irregular nervous action, so prominent a feature in the disease. In hysteria, too, the same effect of the bromide may often be advantageously obtained. So in the treatment of delirium tremens, this remedy, by allaying the extreme erethysm of the nervous centers, produces very decided beneficial effects. In the form or variety of delirium tremens in which there is congestion of the brain, the *delirium*

*ebriosorum* of Solly, the effect of the bromide would be injurious. In such cases it should not be given. In the ordinary cases of delirium tremens, however, when there is inordinate excitement and irritation of the nervous system, the bromide is a remedy of great value.

3. In the irritations and derangements of the nervous system caused by uremic poisoning, we have in the bromide a remedy with which we can often succeed in calming and allaying the irritation and excitement. It does not cause the elimination of the poison, but it counteracts the effects of the poison.

I have prescribed the bromide for several years in typhoid fever, for the purpose of calming the nervous system. Many of the phenomena in typhoid fever depend upon the non-excretion of the solid constituents of the urine—uremic poisoning—and in the bromide we have a useful agent for the relief of the nervous system. It should not be used too long, as I believe that if pushed too far congestion of the brain will be occasioned by it. Skill consists as much in knowing when to suspend, as when to begin the use of a remedy. When the irritation is allayed, then its further use is unnecessary.

4. The bromide will relieve spasm. Several cases of spasmodic stricture of the urethra have been reported relieved by this remedy, given in fifteen to thirty grain doses. I find that Dr. Begbie, of Scotland, and others, report favorably of the use of the bromide in the treatment of asthma.

5. It will soothe and relieve pain. Not as promptly as opium. In some cases opium is inadmissible, however, and then the bromide may sometimes be given to relieve pain. I have relieved several cases of frontal and intercostal neuralgia with the bromide. The bromide is also a very efficient remedy in dysmenorrhœa. By giving this remedy for two or three days before the menstrual period, the pain can often be prevented. It is well to relieve pain. It is better to prevent it.

6. At this time, gentlemen, this is the fashionable remedy for epilepsy. You will find that fashion rules to a considerable extent in our profession. Each period has its fashionable remedy. In the treatment of epilepsy this is the reigning remedy at this time. Permit me to enter my protest against your using this remedy in epilepsy because it is epilepsy. Do not allow the name to suggest the remedy, as the "manner of some is." Epilepsy results from a great variety of conditions. No single remedy will avail. The bromide produces congestion of the brain when given in large doses, or when given in small doses for a considerable time. If you have epilepsy simulated by anemia of the brain, the bromide, by causing blood supply, will relieve the condition. If there be irritation in some of the mucus surfaces, the bromide may give relief If, however, you have a case in which there is congestion of the brain, by giving the bromide you inflict an injury to your patient, and the advice given by Hippocrates, to "avoid doing harm to the patient," is disregarded. Gentlemen, some cases of diseased conditions of the nervous system simulating epilepsy have been, no doubt, relieved by this drug, but I very much doubt whether any case of well defined epilepsy has been permanently cured by it. It will not do to report that a case is permanently cured in three weeks. Such reports are discreditable to the reporter, and an injury to the profession. Thoughtless men—and there are many such—are induced in this way to use a remedy indiscriminately, and often "do harm to their patients."

You will find, gentlemen, that you can promote the effect of the bromide by using it in combination with tincture or fluid extract of valerian. Now and then you will find that nausea and indigestion are induced by this remedy. In such cases you will derive benefit by giving the bromide with syrup, and tincture of gentian, of cinchonæ, or compound tincture of cardamoms.

The bromide of potassium is a valuable remedy when judiciously used, and, like all other remedies of value, when improperly used, it does harm. Ever bear in mind in the use of this and all other remedies, that Hippocrates has said that it is the duty of the physician to do good to the patient and to avoid *doing harm*. Avoid doing harm by not using this remedy in all cases of epilepsy.

